# The Southern Medical College Association and the Four-Years’ Graded Course

**Published:** 1899-01

**Authors:** 


### Selections and Abstracts.

---

### THE SOUTHERN MEDICAL COLLEGE ASSOCIATION AND THE FOUR-YEARS' GRADED COURSE.

During its meeting in Memphis, Tenn., December 5, 1898, this Association unanimously adopted a resolution that all students matriculating in any of their colleges represented in this Association after January 1, 1899, shall be required to attend four full courses of lectures as a requisite to graduation in medicine. Of course, all who matriculated prior to 1899 may graduate in three years, provided the time of graduation be not later than the annual commencements (nearly all of them in the spring) of 1903. The colleges reprented in the act of the Southern Medical College Association on December 5, 1898, are:

| | |
|---|---|
| University of Nashville, | Vanderbilt University, |
| University of Tennessee, | Tennessee Medical College, |
| Memphis Medical College, | Birmingham Medical College, |
| Medical College of Alabama, | Southern Medical College, |
| Tulane University, | University of the South, |
| Texas Medical College. | Medical College of Virginia. |

These, with the colleges of Kentucky that have adopted the four-years' course, compose about two-thirds of the reputable medical colleges of the Southern States.

In connection with this subject, it will be recalled that the American Medical Association, during its session in Denver last May, adopted a resolution which, in effect, takes away from the membership of the Association all those who are connected with colleges which do not, after January 1, 1899, adopt the four-years' graded course. We very much doubt the legality of any such action on the part of the American Medical Association, with such a constitution as it has. It would certainly be unjust, at least, to those members of faculties who really favor the four-years' course, and yet are in the minority in their faculties. But this phase of the subject we do not propose at present to discuss.

This journal has time and again expressed itself as favorable to the four-years' graded course of medical colleges of the country. Our every expression on such a subject since the founding of the *The Virginia Medical Monthly* in 1874 (afterwards changed to *Semi-Monthly*), has been in advocacy of higher medical education so as to perfect, as near as possible, the oncoming generations of doctors. We have neither intention nor desire to recede from such a proposition. There are too many essentials to the perfection of the doctor to be learned in three or even four years of medical college education not to be in general favor of such prolongation of term.

But there are some features connected with the case that do not seem to have been properly considered. In the first place, a natural inquiry is, How did such a question like that decided as it was at Denver, ever come before the American Medical Association? We feel confident that the mass of the profession of America are practically indifferent to the question. It was altogether right, however, and well within the domain of the Association of American Medical Colleges to have advocated such an issue. But when the latter Association, or members thereof, undertook, as they apparently did, to "rush" the matter unexpectedly and without warning through a meeting of the former Association, there is reason to suspect, as a motive for the act, a species of political jobbery or the work of tricksters. The higher, ennobling motives of a generous profession would not have consented to such a "rush-through" method of determining a question that has at least two sides.

The Association of American Medical Colleges is, for the most part, composed of Northern and Northwestern colleges that are heavily endowed. Their faculties are well salaried. But we doubt whether, as teachers, or as men able to judge the merits of the graduate, they are superior to many in the colleges of the Southern States. Competitive examinations of graduates of all the colleges before Army and Navy Boards, or State Medical Examining Boards, etc., are suggestive in this direction.

If the Association of American Medical Colleges has the power to go into the American Medical Association and expel its members, who have for a professional lifetime been honorable men and complying with all of the codal requirements, what limit of authority in the latter Association will the former acquire?

The cost of professional living in most of the great cities of the North and Northwest is much more than in the South. If the political scheming of the Association of American Medical Colleges can accomplish so much as the resolution *rushed* through the American Medical Association was intended to do, it would not surprise us to see at an early day another move, looking to the expulsion from the American Medical Association of any one who is a member of a college faculty that does not adopt some maximum figure as the tuition fee to be required of students. It has just as much right to do the one as the other. Now that the Southern Medical College Association has yielded to the demand for the four-years' course, we caution it to be on its guard lest the major body undertakes to yoke it down to the requirement of charging the same high fees as are charged by many of the colleges of the North. Such fees would, in many instances, rob Southern medical colleges of Southern students.

Let us look at the subject for a moment from another point of view. Many academic colleges, as well as most of the preparatory medical schools, do not claim a rank among graduating medical colleges, although they thoroughly ground their students in microscopy, bacteriology, chemistry and other branches now required of first or second course students in medical colleges. It often happens that such students go to medical colleges as well prepared in these fundamental branches of medical study as are those who have finished like studies in the medical colleges; yet these students or proficients are to get no credit for the work they have accomplished in their academic course. They must, under the compulsory four-years' graded course, spend or lose an entire year in going over studies with which they are already familiar. Such compulsory four-years' course is neither right to the student nor to the college; and it reflects seriously upon the preparatory school. We refer to such instances to show that no inflexible ruling can be made that is just to a large number of students. —*Virginia Medical Semi-Monthly.*

